# Peculiarities of the 7 × 7 to 5 × 5 Superstructure Transition during Epitaxial Growth of Germanium on Silicon (111) Surface

**DOI:** 10.3390/nano13020231

**Published:** 2023-01-04

**Authors:** Vladimir V. Dirko, Kirill A. Lozovoy, Andrey P. Kokhanenko, Olzhas I. Kukenov, Alexander G. Korotaev, Alexander V. Voitsekhovskii

**Affiliations:** Faculty of Radiophysics, National Research Tomsk State University, Lenin Av. 36, 634050 Tomsk, Russia

**Keywords:** 2D materials, two-dimensional structures, molecular beam epitaxy, silicon, germanium, superstructure, reflection high-energy electron diffraction

## Abstract

This paper presents the results of studying the processes of epitaxial growth of germanium on silicon with crystallographic orientation (111) in a wide temperature range. The temperature dependences of the duration of the transition stage from the 7 × 7 to 5 × 5 superstructure and the values of the critical thickness of the transition from two-dimensional to three-dimensional growth in the range from 250 to 700 °C are determined using the reflection high-energy electron diffraction method. It was shown for the first time that the transition time from the 7 × 7 superstructure to 5 × 5 superstructure depends on the temperature of epitaxial growth. The region of low temperatures of synthesis, which has received insufficient attention so far, is also considered.

## 1. Background

The development of epitaxial methods of growth has significantly expanded the production of semiconductor structures with specified parameters. Epitaxial methods have made it possible to create structures with complex profiles and dopant concentrations. An important advantage of epitaxial growth processes is the ability to set the parameters of nanoheterostructures. The control of the heteroepitaxial process opens ways to influence the processes of electric current flow, the dielectric constant, the band gap, the mobility of charge carriers, and the spectrum of electronic states, due to which the characteristics of existing traditional semiconductor devices were improved and new devices based on quantum-size structures were obtained. Nanostructures with quantum wells and quantum dots have been actively used for about a couple of decades in the creation of photodetectors [1,2], solar cells, and light-emitting devices [3] for such rapidly developing areas as nanoelectronics and nanophotonics [4,5,6]. In addition, the prospects of using such structures to create devices of a completely new level, for example, topological transistors, solid-state spin qubits, and highly sensitive sensors, have recently been recognized [7].

An important circumstance in the formation of epitaxial heterostructures is the mismatch of the crystal lattice parameter of the materials used, which affects the elastic stresses in the created system. Due to the complex nature of the behavior of the magnitude and distribution of elastic stresses in the quantum dots/two-dimensional layer system, their directed modification becomes possible in order to create structures for modern straintronics [8,9,10].

To create heterostructures with two-dimensional layers and quantum dots of the best quality, the method of molecular beam epitaxy is used. The presence of a vacuum in the setup makes it possible to place a large amount of analytical equipment for monitoring epitaxial growth, including in situ methods. Interruption of molecular flows occurs due to shutters located on the sources of materials. Due to the above growth conditions, using the method of molecular beam epitaxy, it is possible to create defect-free structures with a thickness from several atomic layers to hundreds of nanometers with sharp heterointerfaces and complex dopant concentration profiles. When using different materials, it is important to take into account the parameters of the lattice constants. Thus, the mismatch of lattice constants between the substrate and the deposited material will lead to the appearance of elastic stresses in the created structure.

The state of the substrate surface has a primary influence on the formation of epitaxial layers. The atoms located on the surface of the substrate have dangling bonds of the crystal lattice, and to reduce the number of these bonds, the surface is reconstructed. On the unreconstructed surface, each top atom in the plane of the surface has several dangling atomic bonds. In reality, after the desorption of foreign oxides and impurities from the surface, the surface is reconstructed in order to reduce the number of dangling bonds. The resulting new bond leads to the appearance on the surface of two bonded atoms—dimers. The formation of dimers leads to a decrease in the surface energy. In the process of epitaxial growth, surface atoms become part of the bulk structure, new deposited atoms become surface atoms, and atomic bonds are constantly reconstructed. The change in energy due to surface reconstruction has a strong effect on the morphology of the growing film [11].

The main factors influencing the ongoing processes are the deposition rate of the material and the substrate temperature. Thus, the process of germanium segregation increases with increasing temperature and is present in the entire range of growth temperatures. With an increase in temperature, the length of migration of adatoms over the surface also increases, and they can reach the edge of the steps, embedding into it. Bulk diffusion with increasing temperature can strongly affect the heterointerface, leading to its degradation and relaxation of stresses created in the structure, and at temperatures above 800 °C, germanium atoms desorb from the surface [12].

Today, the use of the reflection high-energy electron diffraction method is an important analytical component of the molecular beam epitaxy installation under high and ultrahigh vacuum conditions [13]. In the modern sense, the method of diffraction of high-energy reflected electrons is based on the analysis of diffraction patterns obtained by elastic scattering of electrons with energies of 5–100 keV from the surface under study. The method of reflection high-energy electron diffraction has been successfully used to track the growth rate of the epitaxial layer and estimate the number of deposited atomic layers by measuring the intensity oscillations of diffraction patterns [14,15]. The high-energy electron diffraction method is a convenient method for observing epitaxial growth; it is also easily integrated into molecular beam epitaxy installations and does not affect the growth of materials.

Thus, the method of diffraction of high-energy reflected electrons makes it possible to determine the critical thickness of a two-dimensional layer during growth by the Stranski–Krastanov mechanism [16]. During the formation of epitaxial dots, the corresponding reflections appear in the diffraction pattern, analyzing which one can judge the faceting of the islands on the surface. Due to high accelerating voltages, electrons are sensitive to superstructural changes and it is possible to monitor the change in the lattice constant during heteroepitaxial growth. In addition, using the electron diffraction method, the quality of the surface of the test sample is assessed.

Epitaxial nanostructures in the germanium–silicon material system are the most promising in terms of their low cost and the development of technology for creating silicon microcircuits. This pair of materials has been intensively studied for at least 30 years, but the growth temperature range from 400 °C and above is still the most studied, and low-temperature epitaxy has historically received insufficient attention. In particular, the physical mechanism underlying the transition from two-dimensional to three-dimensional growth is still the subject of ongoing scientific discussions [17,18]. Some specific situations, such as formation of quantum dots by low-temperature molecular beam epitaxy [19], after prolonged annealing [20], and without formation of wetting layer [21,22], are also considered in this regard. Usually, the change in the surface energies of the growing material layer [23], as well as the relaxation of accumulated elastic stresses during the formation of nanoislands [24], are mentioned as the reason for this transition. It became obvious that energy considerations alone are not enough to explain the temperature dependence of the critical thickness of the Stranski–Krastanov transition, and deeper considerations of the kinetics of the processes occurring during adsorption, migration, and incorporation of atoms into the growing layer are required [25].

The Si(111) surface was chosen for studying the processes of germanium epitaxial growth due to the fact that this surface is less studied (both experimentally [26,27,28] and theoretically [29,30]) compared to the Si(001) surface [31,32]. However, the surface of pure silicon with the (111) crystallographic orientation is traditionally used for germanium epitaxy [33,34] and is also very promising from the point of view of creating device structures [35,36], especially field-effect transistors [37,38] and photodiodes [39]. Some questions are devoted to Ge dewetting from Si(111) at high temperatures [40], strain relaxation [41], and formation of dislocations [42,43]. Moreover, Si(111) surface is used for epitaxy of GeSn [44] and SiGeSn [45] solid solution, as well as non-group-IV materials, for example, Au [46,47], Ga [48], GaN [49,50], GaSb [51] Bi_2_Te_3_ [52], Se [53], etc. Finally, it is precisely on surfaces with crystallographic orientation (111) that it is possible to create graphene-like two-dimensional materials [48,52,54,55,56], which are highly anticipated for creating electronics of the future [57,58].

This paper presents the results of studies of the transition of the 7 × 7 to 5 × 5 superstructure during the deposition of germanium on silicon substrate with crystallographic orientation (111) with a change in the substrate temperature in the range from 250 to 650 °C. The temperature dependences of the duration of the transition stage from the 7 × 7 to 5 × 5 superstructure and the values of the critical thickness of the transition from two-dimensional to three-dimensional growth in the range from 250 to 700 °C are determined using the reflection high-energy electron diffraction method. It has been shown for the first time that the transition time from the 7 × 7 superstructure to 5 × 5 superstructure depends on the temperature of epitaxial growth. In addition to that, the temperature dependence of the critical thickness of the stressed heteroepitaxial Ge layer and the moment of transition to three-dimensional growth were also determined.

## 2. Experimental Details

Synthesis of Ge on Si(111) substrate was carried out on a Katun-100 high-vacuum molecular beam epitaxy unit. The analytical part of the chamber consists of a mass spectrometer, a quartz thickness monitor, an infrared pyrometer, and 30 keV reflection high-energy electron diffractometer (RHEED). All experiments were carried out at the vacuum level of 1·10^−9^ Torr. Loading of samples was carried out through a loading chamber connected through a vacuum gate. The initial vacuum in the epitaxy chamber and in the loading chamber was created by a vacuum post with Turbo V81M (Varian, Torino, Italy) turbomolecular pump. The ultra-high vacuum was achieved by a 24 h magnetic discharge pump connected to the loading and epitaxy chambers. The final stage was multiple annealing of Ge and Si materials using electron beam evaporator and annealing of the heating element with permanently operating magnetic discharge pump. Auxiliary vacuum equipment was a titan sublimation pump in conjunction with nitrogen cryopanels, which significantly accelerates the processes of degassing.

Commercially available Si(111) wafers with the deviation from the crystallographic plane < 0.5° were used for the studies. Before starting work, the wafers underwent pre-epitaxial preparation, which consisted of chemical treatment and thermal annealing in ultrahigh vacuum. Before placing the wafers into the vacuum chamber, they were subjected to preliminary chemical treatment in order to obtain reaction products with a low evaporation temperature. Chemical cleaning of the silicon wafer consisted of removing the initial oxide layer in a weak solution of hydrofluoric acid, followed by the deposition of a pure oxide layer in a solution of NH_4_OH:H_2_O_2_:H_2_O. At all stages, the plate was washed in a stream of deionized water with a resistance of at least 18 MΩ/cm, followed by drying of the plate. The essence of the method is to chemically create a thin continuous layer of silicon dioxide on the surface of a silicon wafer. This layer has low adsorption capacity with respect to hydrocarbons. After the wafers were placed into the epitaxy installation, the substrate with the protective oxide was heated in the growth chamber at the temperature of 850 °C, which leads to the reduction of the SiO_2_ oxide film to silicon monoxide, which then evaporates from the surface. Hydrocarbons are desorbed at the initial stage of heating, when the silicon dioxide film is still continuous, without interaction with the substrate and formation of carbide particles. Due to the high-quality chemical preparation of the wafer, annealing in a silicon flux, which is often used to clean the substrate, was not used.

**Figure 1:**
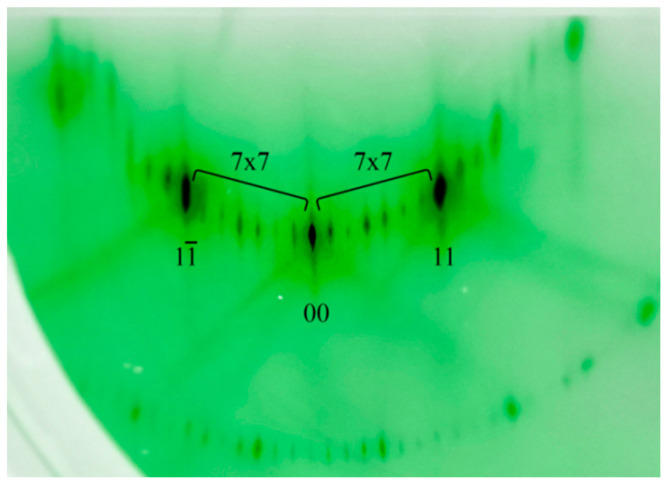
Diffraction pattern from the clean Si(111) surface with the 7 × 7 reconstruction.

During the normal course of the annealing process, a characteristic diffraction pattern with 7 × 7 superstructures for Si(111) is formed on the luminescent screen (Figure 1). In these patterns, elongated spots are main reflections (00) and (11), while short stripes and their number indicate the surface reconstruction. Before epitaxial film growth, silicon buffer layer with thickness > 100 nm is deposited on the wafer surface. The temperature of the silicon substrate during the creation of the buffer is 700 °C. Then, the plate is annealed for 5 min at 1000 °C, after which it is cooled to the required growth temperatures.

The main method for the analysis of superstructural transitions was the method of reflection high-energy electron diffraction. The analysis of diffraction patterns was carried out during epitaxial growth with subsequent computer processing. High resolution of RHEED investigations in our molecular beam epitaxy installation was provided due to high accelerating voltage, stable power supply, low sizes of the diaphragm for electron beam, small incidence angles, low residual gas pressures in the vacuum chamber, and clean RHEED screen. Thus, the method of reflection high-energy electron diffraction can determine superstructural transitions, changes in the lattice parameter of the material, the deposition rate and percentage of solutions, the transition to three-dimensional growth, and the faceting of the resulting epitaxial quantum dots. Diffraction patterns were recorded by a digital video camera with full-HD resolution and a highly sensitive CMOS matrix connected to a computer. Due to the hardware capability of 60 fps camera recording, the software monitors the change in diffraction patterns at speeds up to 60 frames per second. The received video signal is recorded and processed in real time using proprietary algorithms, which makes it possible to measure the change in intensity and the position of spots in diffraction patterns frame by frame.

## 3. Results and Discussion

**Figure 2:**
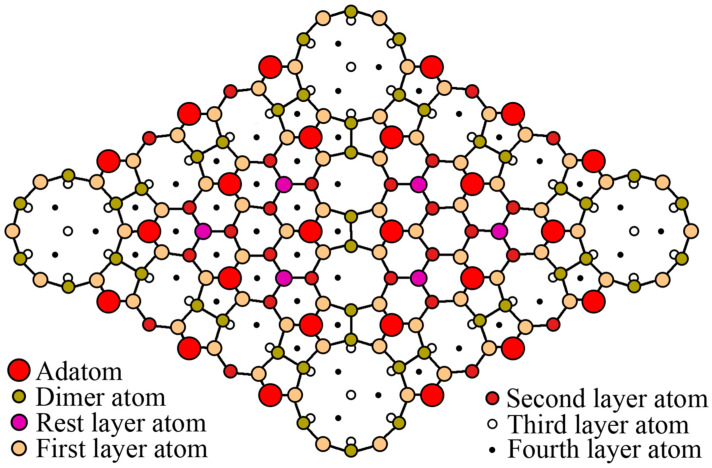
Schematic view of the Ge on Si(111) surface with the 7 × 7 reconstruction.

**Figure 3:**
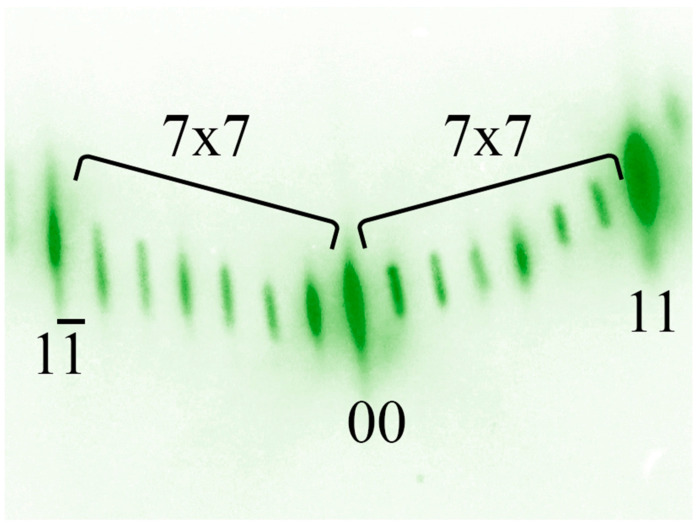
Diffraction pattern from the germanium layer on Si(111) with the 7 × 7 reconstruction at the initial stage of growth.

During the synthesis of Ge on Si(111), the heteroepitaxial system grows according to the Stranski–Krastanov mechanism. A schematic drawing of the Ge on Si(111) surface with the 7 × 7 superstructure is shown in Figure 2. The density of Ge atoms in this structure is 0.72·10^15^ atoms/cm^2^ per one monolayer (ML) of Ge. Therefore, at the beginning of synthesis, the germanium layer repeats the 7 × 7 Si(111) superstructure, as evidenced by the characteristic elongated reflections in the diffraction pattern (Figure 3).

**Figure 4:**
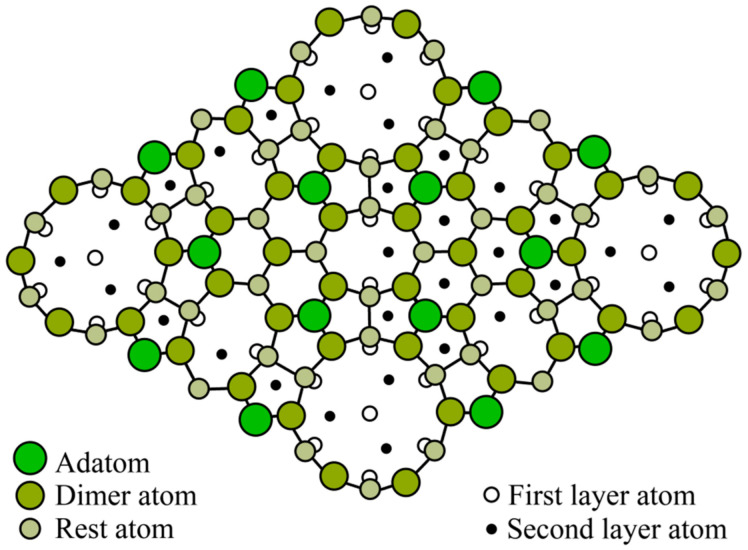
Schematic view of the Ge on Si(111) surface with the 5 × 5 reconstruction.

**Figure 5:**
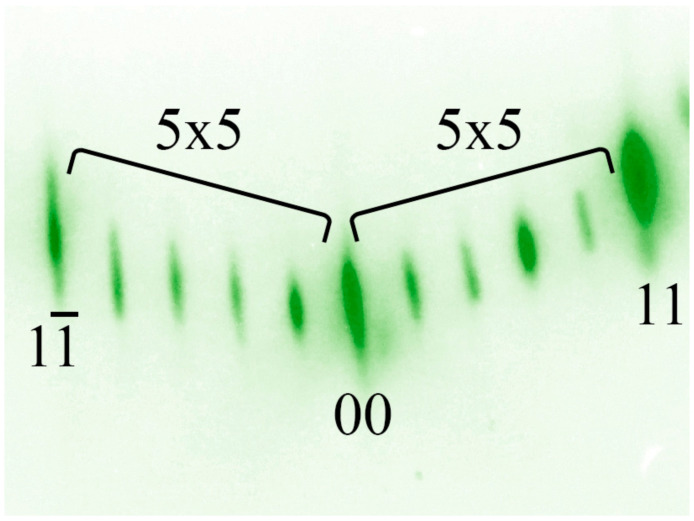
Diffraction pattern from the germanium layer on Si(111) with the 5 × 5 reconstruction at the later stages of growth.

Due to the mismatch of lattice constants between Si and Ge in 4.2%, a change in the arrangement of atoms on the surface and a transition of the 7 × 7 superstructure to the 5 × 5 superstructure occurs. A schematic drawing of the Si(111) surface with the 5 × 5 superstructure is shown in Figure 4, and the corresponding diffraction pattern is shown in Figure 5. The density of Ge atoms in this structure is 0.69·10^15^ atoms/cm^2^ per one monolayer of Ge.

**Figure 6:**
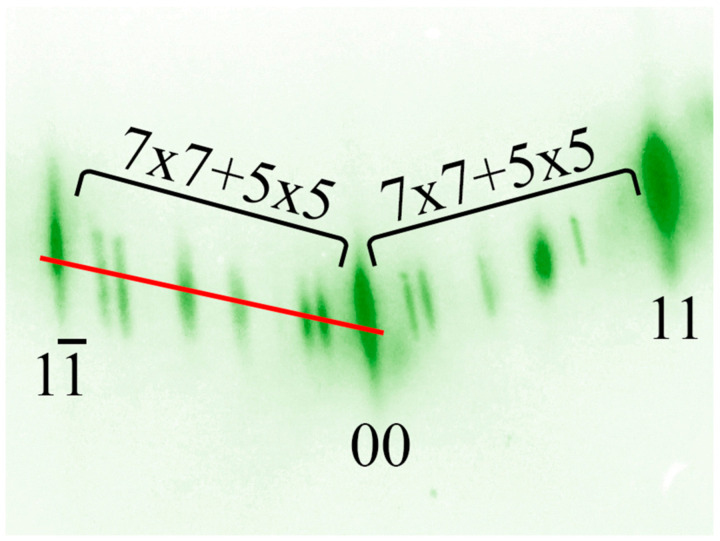
Diffraction pattern from the germanium layer on Si(111) at the moment of the transition from the 7 × 7 to 5 × 5 superstructure. Red line indicates the profile for the subsequent analysis.

**Figure 7:**
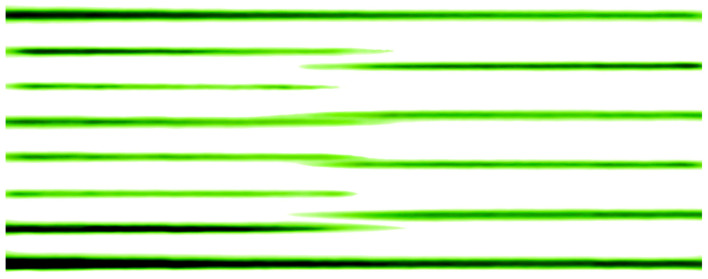
Profile of intensity of diffraction pattern from the germanium layer on Si(111) during the transition from the 7 × 7 to 5 × 5 superstructure.

Cells with the 7 × 7 and 5 × 5 superstructures have different parameters, due to which they are well distinguished in the diffraction pattern (Figure 6). Figure 6 shows a typical diffraction pattern at the time of coexistence of the 7 × 7 and 5 × 5 superstructures. The moment of appearance and disappearance of the superstructure in the diffraction patterns was chosen as the point of intersection of two tangent lines on the intensity curves. Figure 7 shows the profile of changes in diffraction patterns taken during the transition of the 7 × 7 to 5 × 5 superstructure (indicated by the red line in Figure 6) at the substrate temperature of 700 °C and a germanium deposition rate of 0.02 monolayers per second (the thickness of one germanium monolayer on Si(111) is 1.775 Å).

It is clearly seen from this profile that the transition from the 7 × 7 superstructure to the 5 × 5 superstructure is not instantaneous, but can be extended up to a time equal to the duration of the growth of several monolayers. According to the intensity profile of the diffraction pattern, the thickness of the germanium film at the moment of the transition of the 7 × 7 to 5 × 5 superstructure and the percentage coverage of the surface by each superstructure are estimated (Figure 6).

**Figure 8:**
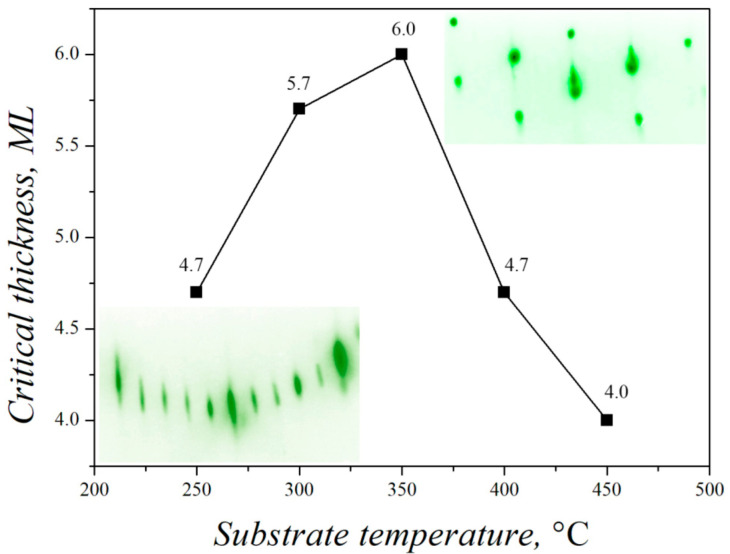
Temperature dependence of the critical thickness on transition from 2D to 3D growth for Ge growth on Si(111).

In addition, by increasing the intensity of the diffraction pattern, one can determine the values of the critical thickness of the 2D germanium layer upon transition to 3D growth depending on the temperature of the Si(111) substrate (Figure 8).

At the temperatures up to 400–450 °C, as the first Ge bilayer grows up, germanium accumulates in the triangular cells of the 7 × 7 superstructure. With the subsequent filling of the first bilayer up to about 50%, two-dimensional islands with a height of one bilayer are generated, and germanium atoms from triangular cells begin to redistribute to the edges of these islands, where there is a deeper potential well for atoms, and, subsequently, two-dimensional islands grow until the full layer is filled [36]. Such an island growth mechanism leads to the fact that at temperatures below 400 °C, strong electron scattering occurs on two-dimensional islands in 7 × 7 cells, and the intensity of diffraction from the 7 × 7 superstructure in the diffraction pattern immediately weakens without the possibility of further analysis. In accordance with Figure 8, the maximum critical thickness of the 2D Ge layer is 5.5–6 ML at the growth temperature of 300–350 °C.

The accumulated stresses during the synthesis of Ge on Si(111) up to 400 °C lead to the formation of 3D islands [59]. The growth of islands occurs not only due to an increase in the lateral size of the island, but also in height, as evidenced by the appearance of a characteristic point diffraction pattern (see inset to Figure 8).

**Figure 9:**
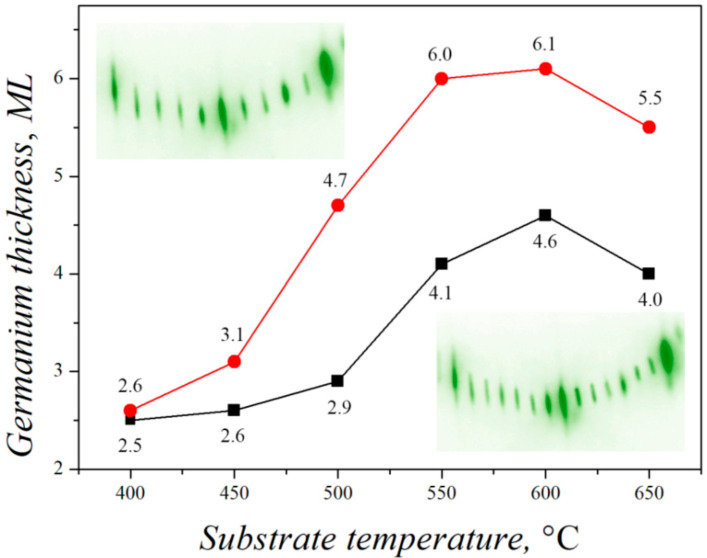
Temperature dependence of the thickness of germanium layer on Si(111) in the moment of transition from the 7 × 7 to 5 × 5 superstructure. Black line: the moment of appearance of the 5 × 5 superstructure. Red line: the moment of disappearance of the 7 × 7 superstructure.

Figure 9 shows the dependence of the germanium film thickness in the moment of transition from the 7 × 7 superstructure to the 5 × 5 superstructure on the temperature of the Si(111) substrate in the range up to 650 °C.

As the germanium epitaxial growth temperature rises, the adatom free path increases and the probability of Ge incorporation at the edges of the steps increases, since there is a deeper potential well for atoms there [36]. The stresses in the system relax due to phase transitions of the 7 × 7 to 5 × 5 superstructure [34,60]. The method of reflection high-energy electron diffraction at the temperatures above 450 °C showed no transition to island growth. The same conclusion, that the surface strain relaxation plays the crucial role in the transition from 7 × 7 to 5 × 5 reconstructions, was also made in a number of previous works, starting from the classical work by Ichikawa and Ino [61], where the processes of transition from 7 × 7 to 5 × 5 reconstructions were studied during high-temperature annealing of the deposited germanium layers. In the present manuscript, we studied the dynamics of these transitions indirectly in the process of germanium atoms deposition with respect to the complicated kinetic nature of the processes on the surface.

At the growth temperatures from ≈400 °C, at the initial stages, germanium adatoms repeat the superstructure of pure Si(111) with the 7 × 7 reconstruction, and with further deposition of germanium, elastic stresses are partially relieved by transition to the 5 × 5 superstructure [62]. It can be seen from Figure 9 that at the temperature of 400 °C, the transition between superstructures occurs fairly quickly at the layer thickness of about 2.5 ML. With an increase in temperature, the thickness of the germanium layer, at which the change of superstructures occurs, increases and at the temperature of 600 °C it reaches 4–6 ML. In this case, the complete transition from one superstructure to another occurs in 1.5–2 atomic layers of germanium, and at the thickness of about 5–5.5 ML the surface is filled with the cells of 7 × 7 and 5 × 5 superstructures in equal proportions. The dependence of the germanium film thickness on the transition from the 7 × 7 superstructure to the 5 × 5 superstructure in Figure 9 can be used to take into account the effect of the substrate temperature on the rate of stress variation in the Ge/Si(111) heteroepitaxial system.

Thus, at low temperatures (up to 450 °C), the growth of Ge on Si(111) proceeds according to the classical Stranski–Krastanov mechanism with the maximum critical thickness of the two-dimensional germanium layer of 6 ML at the temperatures of 300–350 °C and the subsequent formation of 3D islands. At the temperatures above 450 °C, the relaxation of elastic stresses occurs entirely due to 7 × 7–5 × 5 superstructural transitions, and as the substrate temperature increases from 400 to 650 °C, the duration of this process (expressed in the thickness of the deposited material) increases from 0.1 to 2 ML.

## 4. Conclusions

Thus, this paper presents the results of studying the transition of the 7 × 7 to 5 × 5 superstructure during the synthesis of Ge on Si(111) substrate by the reflection high-energy electron diffraction method. The dependences of the duration of the transition of the 7 × 7 to 5 × 5 superstructure at different temperatures of the silicon substrate are obtained. Over the course of the experiments, the temperature dependence of the critical thickness of the stressed heteroepitaxial Ge layer and the moment of transition to three-dimensional growth were also determined.

It was shown that during the epitaxial growth of germanium on silicon with the (111) crystallographic orientation, the range of thicknesses of the deposited two-dimensional layer at which the 7 × 7 to 5 × 5 superstructural transition occurs depends on the growth temperature. In particular, for the Ge deposition rate of 0.04 ML/s, as the substrate temperature increases from 400 to 650 °C, the duration of this process (expressed in the thickness of the deposited material) increases from 0.1 to 2 ML.

The temperature dependences of the parameters of the Ge/Si(111) system obtained by reflection high-energy electron diffraction can be used in modeling the synthesis of GeSi/Si structures [63,64,65], as well as for further development of the technological process of fabricating materials by molecular beam epitaxy for creation of optoelectronic devices and nanoelectronics based on Ge/Si and GeSi/Si nanoheterostructures [66]. In addition, the results obtained can clarify the nature of surface processes and the physical mechanism of the transition from two-dimensional to three-dimensional growth in other mismatched epitaxial systems.

## Data Availability

The authors declare that the data supporting the findings of this study are available within the article.
